# Supplementary material for: The palmitoyl acyltransferase ZDHHC14 controls Kv1-family potassium channel clustering at the axon initial segment
Source: eLife. 2020 Nov 13;9:e56058. doi: 10.7554/eLife.56058 (PMC7685708; doi:10.7554/eLife.56058)
Supplement: Transparent reporting form [file elife-56058-transrepform.pdf]

1st Floor
24 Hills Road
Cambridge CB2 1JP, UK

P 01223 855340
W elifesciences.org
T @elife

eLife's transparent reporting form

We encourage authors to provide detailed information *within their submission* to facilitate the interpretation and replication of experiments. Authors can upload supporting documentation to indicate the use of appropriate reporting guidelines for health-related research (see EQUATOR Network), life science research (see the BioSharing Information Resource), or the ARRIVE guidelines for reporting work involving animal research. Where applicable, authors should refer to any relevant reporting standards documents in this form.

If you have any questions, please consult our Journal Policies and/or contact us: editorial@elifesciences.org.

Sample-size estimation

- You should state whether an appropriate sample size was computed when the study was being designed
- You should state the statistical method of sample size computation and any required assumptions
- If no explicit power analysis was used, you should describe how you decided what sample (replicate) size (number) to use

Please outline where this information can be found within the submission (e.g., sections or figure legends), or explain why this information doesn't apply to your submission:

No statistical method was used to compute sample sizes while the study was being designed. However, the sample sizes used in each experiment were based on similar prior studies from our group (Thomas GM et al, Neuron, 2012; George J et al, Elife, 2015) and others (Ogawa et al, J Neurosci, 2008) that assessed similar readouts.

Replicates

- You should report how often each experiment was performed
- You should include a definition of biological versus technical replication
- The data obtained should be provided and sufficient information should be provided to indicate the number of independent biological and/or technical replicates
- If you encountered any outliers, you should describe how these were handled
- Criteria for exclusion/inclusion of data should be clearly stated
- High-throughput sequence data should be uploaded before submission, with a private link for reviewers provided (these are available from both GEO and ArrayExpress)

Please outline where this information can be found within the submission (e.g., sections or figure legends), or explain why this information doesn't apply to your submission:

The exact number and type of replicates for each experiment is reported in the corresponding figure legend and in the source data file and clarification regarding definition of biological/technical replicates is provided in the following methods sections: “Western blot and quantification”, “Image acquisition and analysis”, and “Electrophysiological recordings”. No outliers were excluded. Criteria to limit analysis to intact Axon Initial Segments (AISs) and minimize contribution from multiple overlapping AISs, as well as inclusion criteria for imaging transfected neurons are described in the “Image acquisition and analysis” section of the methods.

Statistical reporting

- Statistical analysis methods should be described and justified
- Raw data should be presented in figures whenever informative to do so (typically when N per group is less than 10)
- For each experiment, you should identify the statistical tests used, exact values of N, definitions of center, methods of multiple test correction, and dispersion and precision measures (e.g., mean, median, SD, SEM, confidence intervals; and, for the major substantive results, a measure of effect size (e.g., Pearson's r, Cohen's d)
- Report exact p-values wherever possible alongside the summary statistics and 95% confidence intervals. These should be reported for all key questions and not only when the p-value is less than 0.05.

Please outline where this information can be found within the submission (e.g., sections or figure legends), or explain why this information doesn’t apply to your submission:

The statistical analysis method used for each experiment is indicated in the corresponding figure legend along with exact N, test corrections, confidence intervals, and, when possible, exact p values and effect sizes. As stated in the Methods sections, mean is indicated on all graphs and error bars indicate standard deviation: “Western blot and quantification”, “Image acquisition and analysis”, and “Electrophysiological recordings”. When appropriate, data are presented with the raw data shown as scatter plots.

(For large datasets, or papers with a very large number of statistical tests, you may upload a single table file with tests, Ns, etc., with reference to sections in the manuscript.)

Group allocation

- Indicate how samples were allocated into experimental groups (in the case of clinical studies, please specify allocation to treatment method); if randomization was used, please also state if restricted randomization was applied
- Indicate if masking was used during group allocation, data collection and/or data analysis

Please outline where this information can be found within the submission (e.g., sections or figure legends), or explain why this information doesn’t apply to your submission:

In all cases, plates or wells of cells were randomly assigned to the different testing groups as described in the "Western blot and quantification", "Image acquisition and analysis", and "Electrophysiological recordings" methods sections. No masking was used during group allocation or for data collection in biochemical experiments. However, for the majority of imaging experiments the experimenter first selected a field of interest based on AnkG fluorescent signal, with no knowledge of the experimental signal (PSD93, Kv1.1-1.4) that was subsequently acquired in a separate channel. No masking was used during data analysis.

Additional data files ("source data")

- We encourage you to upload relevant additional data files, such as numerical data that are represented as a graph in a figure, or as a summary table
- Where provided, these should be in the most useful format, and they can be uploaded as "Source data" files linked to a main figure or table
- Include model definition files including the full list of parameters used
- Include code used for data analysis (e.g., R, MatLab)
- Avoid stating that data files are "available upon request"

Please indicate the figures or tables for which source data files have been provided:

All quantified numerical data are provided in the "source data" Excel files, which are organized by figure number.
